# Kappa Opioid Receptor Blockade in the Amygdala Mitigates Pain Like-Behaviors by Inhibiting Corticotropin Releasing Factor Neurons in a Rat Model of Functional Pain

**DOI:** 10.3389/fphar.2022.903978

**Published:** 2022-05-25

**Authors:** Vadim Yakhnitsa, Guangchen Ji, Matthew Hein, Peyton Presto, Zack Griffin, Olga Ponomareva, Edita Navratilova, Frank Porreca, Volker Neugebauer

**Affiliations:** ^1^ Department of Pharmacology and Neuroscience, Texas Tech University Health Sciences Center, Lubbock, TX, United States; ^2^ Center of Excellence for Translational Neuroscience and Therapeutics, Texas Tech University Health Sciences Center, Lubbock, TX, United States; ^3^ Department of Pharmacology, College of Medicine, University of Arizona, Tucson, AZ, United States; ^4^ Garrison Institute on Aging, Texas Tech University Health Sciences Center, Lubbock, TX, United States

**Keywords:** amygdala, functional pain syndrome, corticotropin-releasing factor, kappa opioid receptor, plasticity, behavior

## Abstract

Functional pain syndromes (FPS) occur in the absence of identifiable tissue injury or noxious events and include conditions such as migraine, fibromyalgia, and others. Stressors are very common triggers of pain attacks in various FPS conditions. It has been recently demonstrated that kappa opioid receptors (KOR) in the central nucleus of amygdala (CeA) contribute to FPS conditions, but underlying mechanisms remain unclear. The CeA is rich in KOR and encompasses major output pathways involving extra-amygdalar projections of corticotropin releasing factor (CRF) expressing neurons. Here we tested the hypothesis that KOR blockade in the CeA in a rat model of FPS reduces pain-like and nocifensive behaviors by restoring inhibition of CeA-CRF neurons. Intra-CeA administration of a KOR antagonist (nor-BNI) decreased mechanical hypersensitivity and affective and anxiety-like behaviors in a stress-induced FPS model. In systems electrophysiology experiments in anesthetized rats, intra-CeA application of nor-BNI reduced spontaneous firing and responsiveness of CeA neurons to peripheral stimulation. In brain slice whole-cell patch-clamp recordings, nor-BNI increased feedforward inhibitory transmission evoked by optogenetic and electrical stimulation of parabrachial afferents, but had no effect on monosynaptic excitatory transmission. Nor-BNI decreased frequency, but not amplitude, of spontaneous inhibitory synaptic currents, suggesting a presynaptic action. Blocking KOR receptors in stress-induced FPS conditions may therefore represent a novel therapeutic strategy.

## 1 Introduction

Functional pain syndromes (FPS) are chronic illnesses that can be aggravated or precipitated by repeated stress. FPS differ from most pain conditions in that they are not associated with identifiable tissue injury or pathology. FPS include conditions such as fibromyalgia, migraine, chronic fatigue syndrome, medication overuse syndrome, and others (Kim and Chang, 2012; Bettini and Moore, 2016). Patients with FPS experience intermittent episodic pain attacks often triggered by stress. Repeated stressors lead to a transition from episodic pain to chronic pain. Kim and Chang, (2012); Gulewitsch et al. (2017); Dodick, (2018).

Dynorphin, an endogenous agonist at kappa opioid receptors (KOR), plays a critical role in aversive behavioral responses to stressors (Knoll and Carlezon, 2010). KOR are involved in numerous behavioral aversive effects such as dysphoria, anhedonia, increased anxiety and depression (Feder et al., 2009; Knoll and Carlezon, 2010; Crowley et al., 2016). Systemic administration of a KOR antagonist has anxiolytic effects in the elevated plus maze test (Knoll et al., 2007). KOR are extensively expressed throughout limbic brain areas including the central nucleus of amygdala (CeA), the basolateral amygdala (BLA), extended amygdala, hippocampus and hypothalamus (Fallon and Leslie, 1986; Mansour et al., 1995; Hurd, 1996; Alheid, 2003; Nestler and Carlezon, 2006; Koob, 2008; Schwarzer, 2009; Cahill et al., 2014).

The CeA plays an important role in emotional-affective responses, development of aversive behaviors, stress-related disorders, and pain modulation (Neugebauer et al., 2004; Neugebauer, 2015, Neugebauer, 2020). Dynorphin (endogenous KOR ligand) is synthesized in neurons in the lateral CeA, a subset of which are corticotropin-releasing factor (CRF) neurons (Marchant et al., 2007). Many CeA-CRF neurons also co-express dynorphin precursor, prodynorphin (Pomrenze et al., 2015), suggesting an interaction of CRF and KOR systems in the amygdala. Nociceptive information from spinal cord and brainstem reaches CeA-CRF neurons through peptidergic afferent input from the parabrachial nucleus (Harrigan et al., 1994; Han et al., 2010) and in turn, the CeA is the major source of CRF-containing projection pathways to the extra-amygdalar targets promoting aversive and anxiety-like behaviors (Fendt et al., 1997; Marcilhac and Siaud, 1997; Beckerman et al., 2013; McCall et al., 2015; Pomrenze et al., 2015; Pomrenze et al., 2019a; Pomrenze et al., 2019b). Increased activity of CeA-CRF neurons by KOR agonist promotes averse-affective pain-like behaviors through synaptic disinhibition in naïve rats (Hein et al., 2021).

Increasing evidence suggests that KOR signaling is involved in pain processing in the CeA (Neugebauer et al., 2020). Under normal conditions, KOR activation in the CeA has pronociceptive effects by decreasing feedforward inhibitory transmission and increasing evoked and spontaneous activity of CeA neurons (Ji and Neugebauer, 2020; Hein et al., 2021). Pretreatment with a KOR antagonist (nor-binaltorphimine, nor-BNI) prevented conditioned place preference (CPP) to intravenous gabapentin in the spinal nerve ligation model (SNL) of neuropathic pain, which was interpreted to suggest that nor-BNI removed the aversiveness of ongoing pain. At the cellular level, nor-BNI decreased synaptically evoked spiking of CeA neurons recorded in brain slices from SNL rats but not from sham controls (Navratilova et al., 2019).

The role of KOR signaling in FPS is only beginning to emerge. Several rodent models of FPS have been developed recently (Xie et al., 2017; Nation et al., 2018; Kopruszinski et al., 2021; Navratilova et al., 2021). FPS models use a “two-hit” hyperalgesic priming strategy in which a priming stimulus (i.e., nociceptive input) is followed by a normally subthreshold stimulus, resulting in exaggerated pain response. In the current study, we have adapted this strategy in rats using priming with morphine (i.e., the first hit). Following priming, stress (i.e., the second hit) effectively promotes pain responses, consistent with clinical observations of FPS. This model was characterised in preliminary form (Ji et al., 2019). Studies in FPS models demonstrated that intra-CeA administration of nor-BNI prevented stress-induced mechanical hypersensitivity allodynia in an injury free model of medication overuse (Xie et al., 2017) and also prevented stress-induced loss of diffuse noxious inhibitory control (DNIC) in morphine-primed rats (Nation et al., 2018). Under normal conditions, intra-CeA KOR blockade had no effect on mechanical sensitivity or DNIC (Xie et al., 2017). The data suggest that KOR activation enhanced descending facilitation in pain conditions. Increase in dynorphin release and/or increased KOR function (phosphorylation) could promote the changes in KOR signaling observed in FPS. (Bruchas et al., 2009; Xie et al., 2017).

While KOR antagonists may provide a new strategy for the management of stress-related functional pain disorders, their mechanisms of actions are not completely understood. This study examined the role of KOR signaling in a rat model of FPS on pain-like and averse-effective behaviors. We tested the hypothesis that blockade of KOR signaling by a selective KOR antagonist in the CeA increases synaptic inhibition of CeA-CRF neurons driven by parabrachial input to CeA.

## 2 Results

This study tested the hypothesis that blockade of KOR signaling in the CeA would mitigate pain-like behaviors in an FPS rat model through inhibition of CRF neurons in the CeA. Behavioral assays, systems electrophysiology and brain slice electrophysiology were used to assess the effects of a selective long-lasting KOR antagonist (nor-binaltorphimine, nor-BNI) on CeA neurons. Since CRF is important in pain-related amygdala plasticity, we employed genetic approaches to label CeA-CRF neurons and to test effect of nor-BNI on their spontaneous and evoked activity.

### 2.1 Functional Pain Priming Model

**FIGURE 1:**
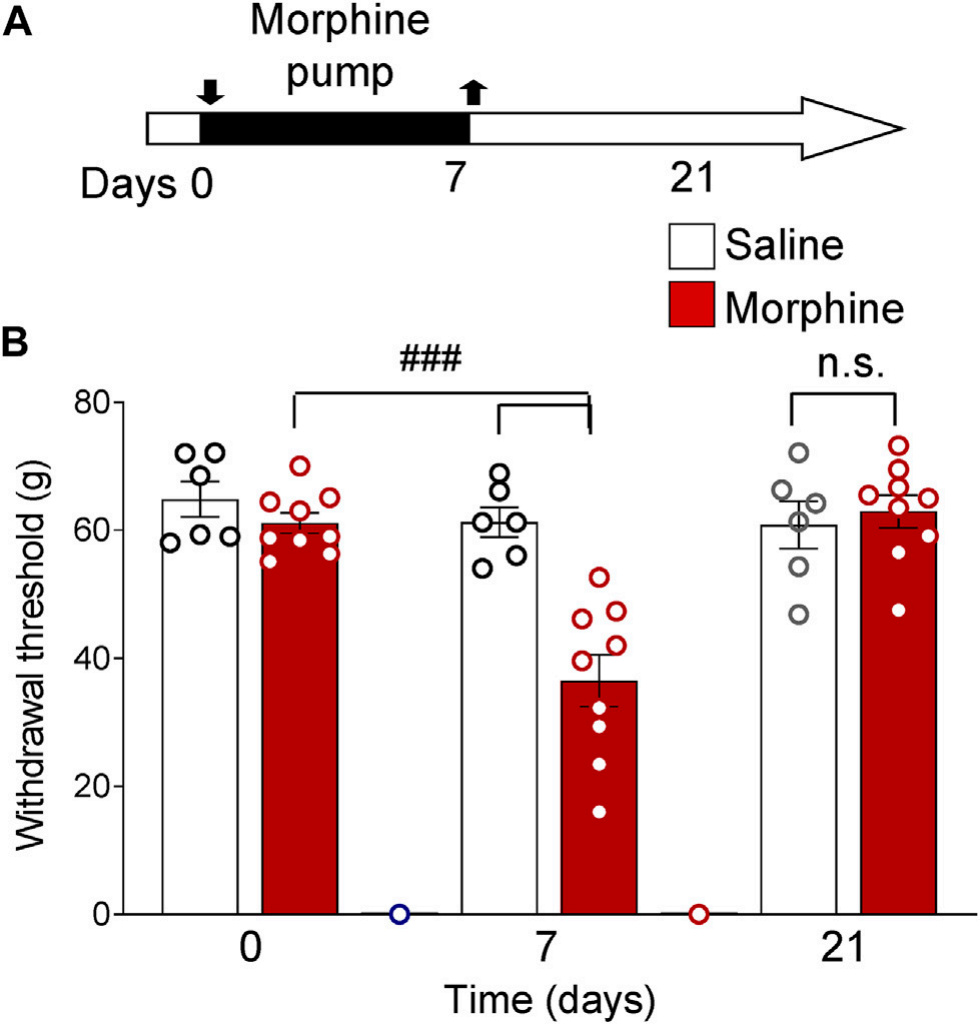
Functional priming pain model. Increased mechanosensitivity induced by morphine treatment (priming). **(A)** Timeline of experimental procedures. Rats were treated with morphine or saline for 7 days. **(B)** Morphine-treated rats showed decreased paw withdrawal thresholds compared to morphine-treated baseline measures and compared to saline group on day 7 (^###^ *p* < 0.001, two-way ANOVA with Tukey’s posthoc test, *n* = 15). By day 21 mechanosensitivity recovered to baseline levels (n.s., *p* > 0.05, two-way ANOVA with Tukey’s posthoc test, *n* = 15). Bar histograms show means ± SEM. Symbols represent values of individual rats.

**FIGURE 2:**
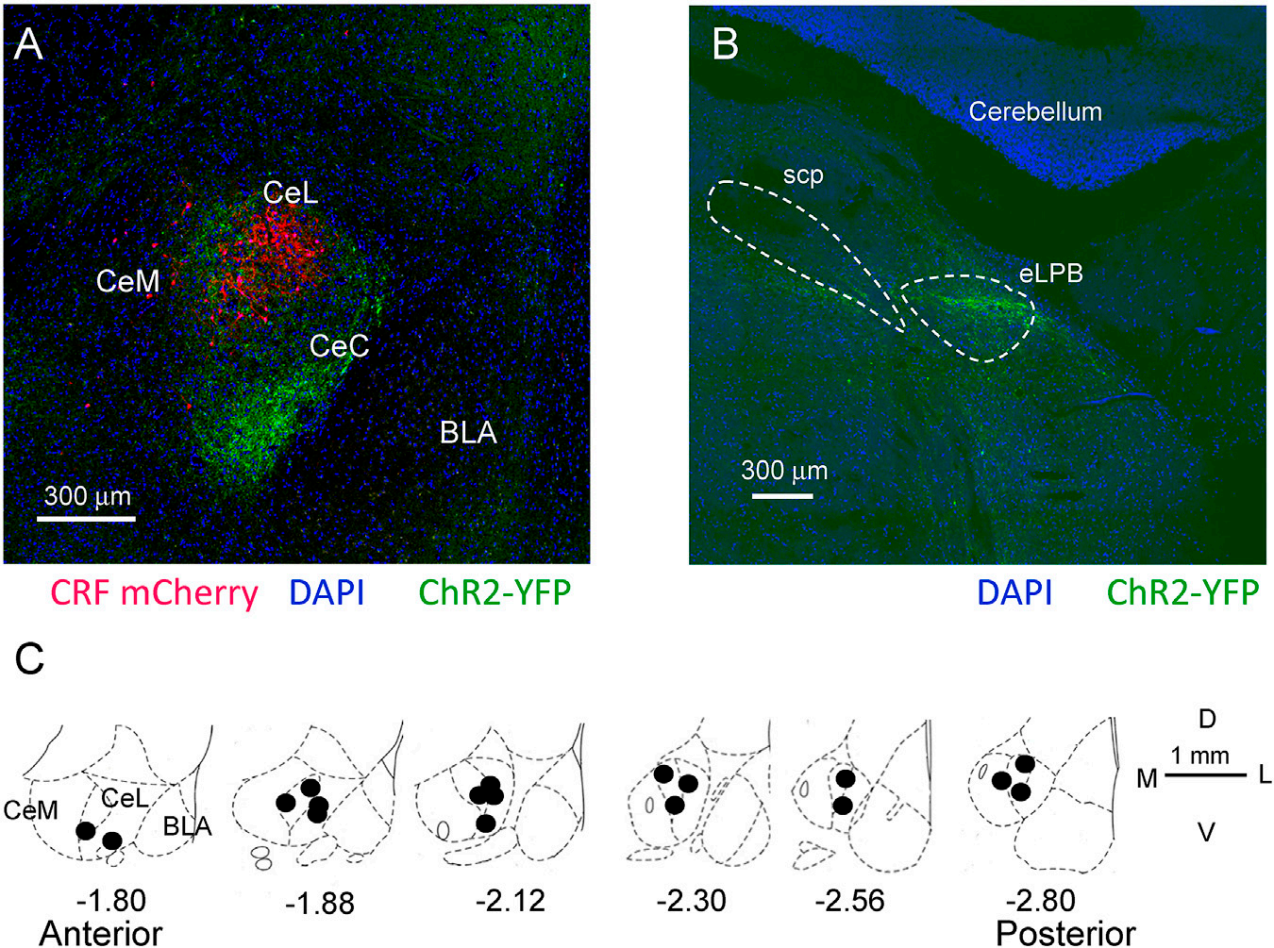
Location of CRF neurons, ChR2 injections for optogenetics, and microdialysis probes in the CeA. **(A)** Confocal image of mCherry labeled CRF neurons in a Crh-Cre rat 5 weeks post viral (AAV5- EF1α-DIO-mCherry, red) injection into CeA. Glutamatergic terminals expressing channelrhodopsin 2 (ChR2-eYFP) 5 weeks post viral injection (rAAV5/CaMKIIa-ChR2(H134R)-eYFP, green) into the external parabrachial nucleus (PB). CeM, CeL, CeC, medial, lateral, and capsular divisions of amygdala; BLA, basolateral amygdala. **(B)** Injection site for rAAV5-CaMKIIa-hChR2eYFP in the external lateral PB for ChR2 expression in glutamatergic neurons. SPC, superior cerebellar peduncle. **(C)** Diagrams show coronal brain slices at different anterior-posterior levels. Filled circles indicate the positions of the microdialysis probe tips for drug application into CeA that were included in this study.

Treatment of rats with saline for 7 days (timeline in Figure 2A) had no significant effect on sensory withdrawal thresholds in both hind paws measured with a plantar electronic von Frey anesthesiometer. Priming with morphine for 7 days significantly decreased withdrawal thresholds (*p* < 0.0001, two-way ANOVA, F_2,39_ = 13.8, Tukey’s posthoc test, *n* = 15, interaction *p* < 0.001, F_2,39_ = 10.67, Figure 1). In the morphine-treated group, withdrawal thresholds recovered by day 21 and were not significantly different from saline group thresholds (*p* > 0.05, two-way ANOVA with Tukey’s posthoc test, Figure 1).

### 2.2 Inhibitory Behavioral Effects of Intra-Central Nucleus of Amygdala Kappa Opioid Receptors Antagonist in Functional Pain Syndromes

**FIGURE 3:**
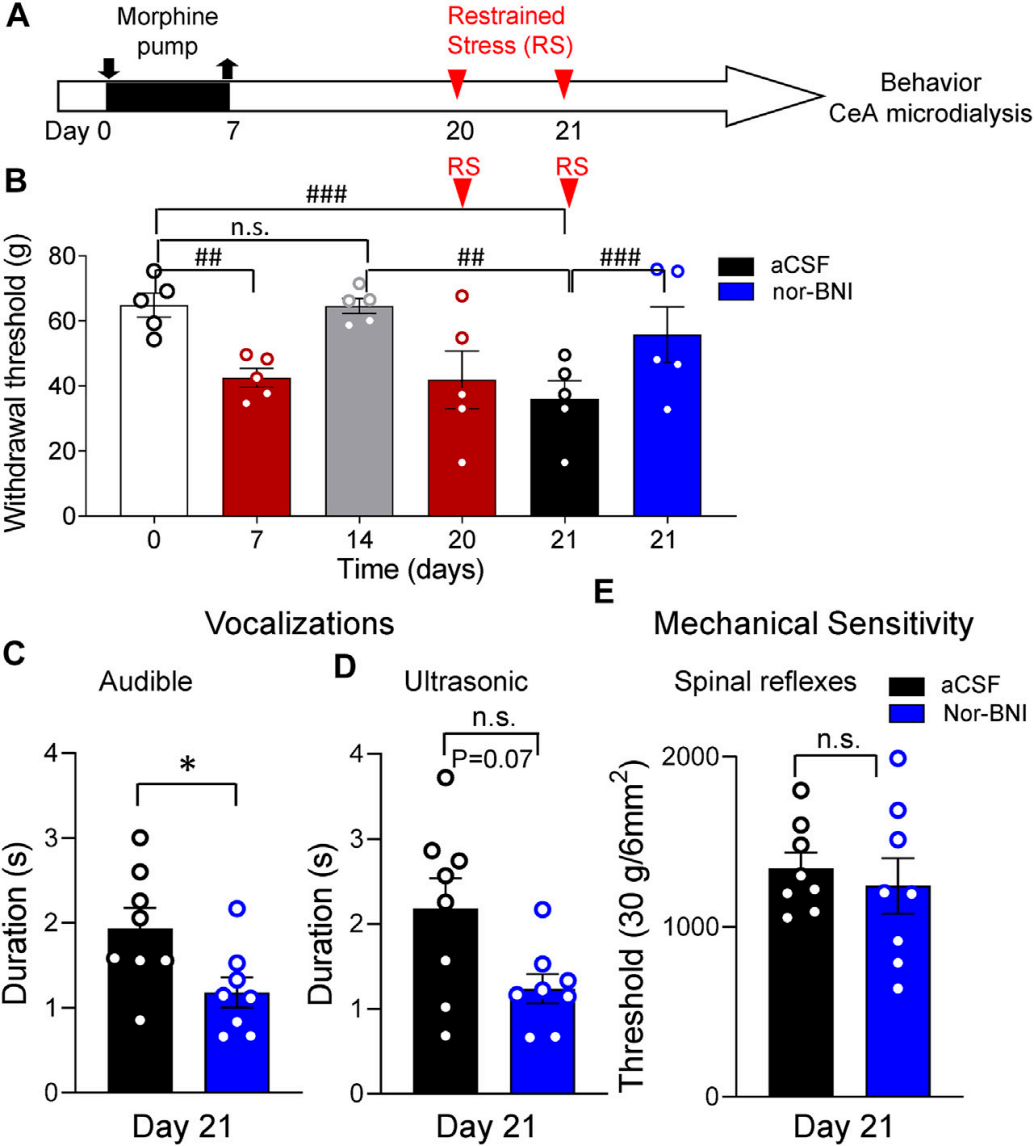
Inhibitory effects of intra-CeA KOR antagonist (nor-BNI) on pain-like and affective behaviors in a functional pain model. **(A)** Timeline of experimental procedures. Rats were treated with morphine for 7 days. Restrained stress (RS) was induced on days 20 and 21. **(B)** Morphine priming decreased paw withdrawal thresholds measured with electronic von Frey on day 7 (^##^ *p* < 0.01, ANOVA RM, Tukey’s posthoc test, *n* = 5). By day 14 mechanosensitivity recovered to baseline levels (n.s., *p* > 0.05, ANOVA RM, with Tukey’s posthoc test, *n* = 5). RS on day 21 paw significantly decreased withdrawal thresholds compared to day 0 and day 14 (^#^ *p* < 0.05, ANOVA RM, with Tukey’s posthoc test, *n* = 5). Nor-BNI (100 μM in microdialysis probe) administered into the CeA significantly increased withdrawal thresholds compared to vehicle (aCSF) (*p* < 0.001, ANOVA RM, Tukey’s posthoc test, *n* = 5). **(C,D)** After RS on day 21, nor-BNI significantly increased audible vocalizations **(C)** in response to noxious compression of the hind paw (*p < 0.05, paired *t*-test, compared to aCSF control, *n* = 8). Decrease in ultrasonic vocalizations after intra-CeA application of nor-BNI did not reach level of significance **(D)** (n.s. *p =* 0.07, paired *t*-test, compared to aCSF control, *n* = 8). **(E)** Intra-CeA administration of nor-BNI had no significant effect on noxious mechanical withdrawal thresholds evoked by compression of the hind paw. (n.s. *p >* 0.05, paired *t*-test, compared to aCSF, *n* = 8). Bar histograms show means ± SEM. Symbols represent values of individual rats.

Another group of rats was subjected to morphine priming followed by repeated restrained stress (RS, timeline in Figure 3A). After a 7-day treatment with morphine, rats developed mechanical hypersensitivity measured with von Frey anesthesiometer [*p* < 0.01, repeated-measures (RM) ANOVA, F_5,20_ = 14.33, Tukey’s posthoc test, *n* = 5, Figure 3B]. Sensitivity recovered to baseline levels by day 14 (*p* > 0.05, RM ANOVA with Tukey’s posthoc test, Figure 3B). One-hour RS on day 20 significantly decreased withdrawal thresholds compared to day 0 and day 14 (*p* < 0.01, RM ANOVA with Tukey’s posthoc test, Figure 3B). Successive RS on day 21 further decreased withdrawal thresholds (*p* < 0.001, RM ANOVA with Tukey’s posthoc test, Figure 3B). Administration of a KOR antagonist (nor-BNI, 100 μM) into the CeA (Figure 2A) significantly increased withdrawal thresholds compared to vehicle control (aCSF) (*p* < 0.001, RM ANOVA with Tukey’s posthoc test, Figure 3B). After 15 min of nor-BNI microdialysis, mechanosensitivity measured with von Frey anesthesiometer was not significantly different from baseline (pre-priming) values (*p* > 0.05, RM ANOVA with Tukey’s posthoc test, Figure 3B).

Intra-CeA microdialysis of nor-BNI also significantly decreased emotional-affective responses measured as the duration of audible vocalizations in response to brief (15 s) noxious compression of the hindpaw (Section 5.5.2; *p* < 0.05, paired *t*-test, *n* = 8; Figure 3C). Durations of ultrasonic vocalizations also decreased but did not reach a significant level (*p* = 0.07, paired *t*-test, *n* = 8; Figure 3D). In contrast to withdrawal thresholds evoked with von Frey anesthesiometer (Figure 3B), withdrawal thresholds evoked by hindpaw compression were not changed by nor-BNI microdialysis into the CeA (*p* > 0.05, paired *t*-test, *n* = 8; Figure 3E).

**FIGURE 4:**
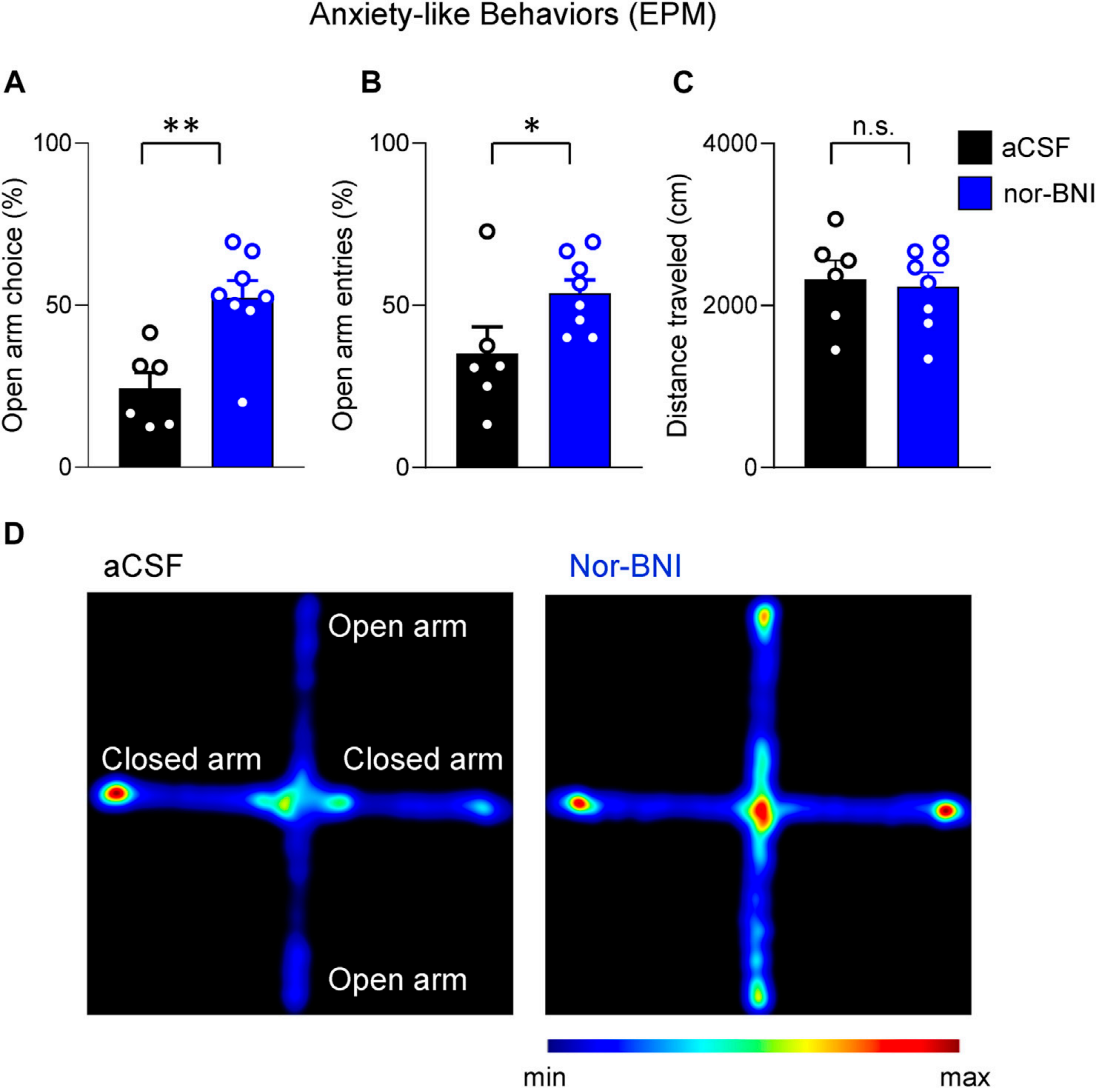
Inhibitory effects of intra-CeA KOR antagonist (nor-BNI) on anxiety-like behaviors in a functional pain model. Intra-CeA nor-BNI (100 μM in microdialysis probe) increased percentage of time in open arms **(A)** and frequency (percent of total number) of entries into open arms **(B)** of the elevated plus maze (EPM), indicating anxiety-like behaviors. *, **p < 0.05, 0.01, *t*-tests, compared to aCSF control, *n* = 6 (aCSF), *n* = 8 (nor-BNI). Total distance travelled was not different between control (aCSF) and treatment (nor-BNI) groups **(C)** (n.s., *p* > 0.05, *t*-test, *n* = 6 (aCSF), *n* = 8 (nor-BNI). Bar histograms show means ± SEM. Symbols represent values of individual rats. **(D)** Representative heat maps show time spent in the closed and open arms of the EPM. A rat pretreated with aCSF into the CeA spent more time in the closed arm (left panel), whereas a nor-BNI pretreated rat spent equal time in the closed and open arms (right panel).

Nor-BNI also decreased anxiety-like behaviors in the elevated plus maze test (EPM), which was measured as time spent by an animal in the open arms (*p* < 0.01, unpaired *t*-test, *n* = 5, aCSF, *n* = 8, nor-BNI; Figure 4A) and as number of entries into open arms (*p* < 0.05, unpaired *t*-test, Figure 4B). These changes were not related to motor deficits because total distance traveled over the 5 min time period did not change in both groups of animals (aCSF and nor-BNI) (*p* > 0.05, unpaired *t*-test, Figure 4C). As seen on heatmaps, exploratory behavior of rats in the EPM was strongly facilitated by intra-CeA nor-BNI as compared to aCSF (Figure 4D).

The data suggest that in morphine-primed rats, activation of KOR in the CeA is stress-dependent. Inhibition of intra-CeA KOR signaling reduces nocifensive, affective, and anxiety-like behaviors in a rat model of FPS.

### 2.3 Inhibitory Effects of a Kappa Opioid Receptors Antagonist on Central Nucleus of Amygdala Neurons in Functional Pain Syndromes. Systems Electrophysiology

**FIGURE 5:**
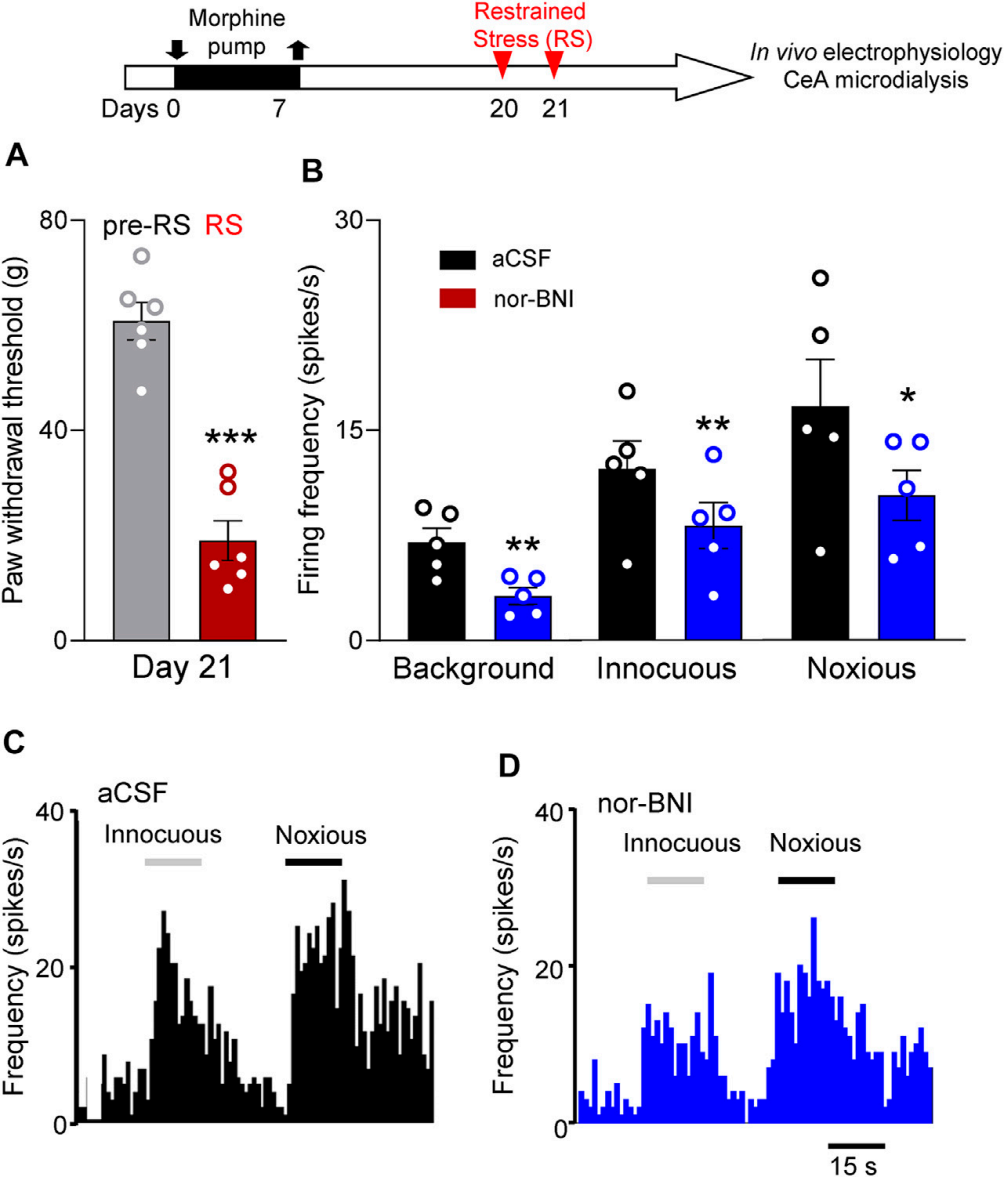
Intra-amygdalar KOR antagonist decreased activity of CeA neurons in a functional pain model. Timeline of experimental procedures is shown at the top of Figure. **(A)** Restrained stress (RS) decreased paw withdrawal thresholds (***p < 0.001, paired *t*-test, *n* = 6 rats) measured with electronic von Frey in morphine-primed rats used in the electrophysiology study **(B–D)**. **(B)** Administration of nor-BNI (100 μM in microdialysis probe) into CeA decreased background activity (**p < 0.01, *n* = 5 neurons) of CeA neurons and responses to innocuous and noxious stimulations (*, **p < 0.05, 0.01, *n* = 5 neurons) compared with predrug control values (aCSF). Bar histograms show means ± SEM. Symbols show values for individual rats **(A)** and neurons **(B)**.

Individual CeA neurons were recorded in morphine-primed rats subjected to repeated RS (Timeline, Figure 5). Repeated RS significantly decreased withdrawal thresholds measured with von Frey anesthesiometer in these rats (*p* < 0.001, paired *t*-test, *n* = 6, Figure 5A). After RS (2–3 h window), activity of CeA neurons was recorded extracellularly in anaesthetized animals. One neuron was recorded in each rat while vehicle (aCSF) or nor-BNI was administered by microdialysis (nor-BNI, 100 μM, 15 min). Only neurons who responded more strongly to noxious than innocuous stimulation were selected as described previously (Ji and Neugebauer, 2014; Ji and Neugebauer, 2020). Nor-BNI significantly decreased background activity and neuronal responses evoked by innocuous and noxious mechanical compression of the hindpaw with a calibrated forceps (Sections 5.5.2 and 5.6.1; *p* < 0.01 and *p* < 0.05, respectively, compared to vehicle aCSF, paired *t*-test, *n* = 5; Figure 5B).

### 2.4 Inhibitory Effects of a Kappa Opioid Receptors Antagonist on Central Nucleus of Amygdala-Corticotropin Releasing Factor Neurons in Functional Pain Syndromes. Brain Slice Electrophysiology

**FIGURE 6:**
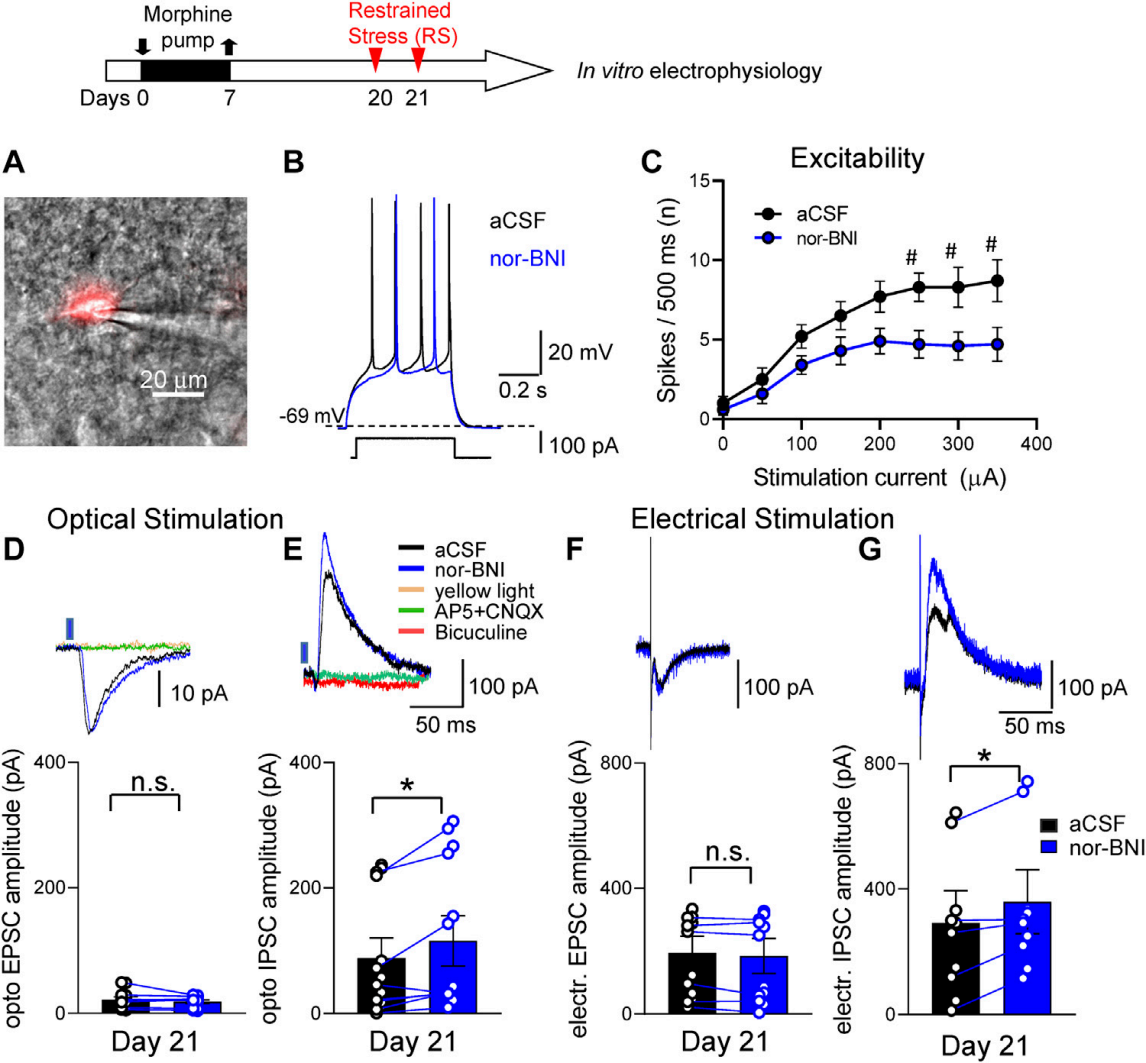
Effects of a KOR antagonist on excitatory and inhibitory synaptic transmission in CRF-CeA neurons in a functional pain model. Timeline of experimental procedures is shown at the top of the Figure. Recordings of visually identified CeA-CRF neurons were made in whole-cell configuration. **(A)** A CRF-CeA neuron is shown with fluorescence (for mCherry) illumination. **(B)** Traces of a late-firing neuron (shown in **A**) during aCSF and nor-BNI (1 μM) application. Nor-BNI decreased neuronal firing evoked by depolarizing current injection. **(C)** Application of nor-BNI decreased excitability (**F–I** relationship) of CeA-CRF neurons in brain slices from rats with functional pain syndrome model (^#^ *p* < 0.05, ANOVA RM, Sidak’s posthoc test, *n* = 10). **(D,F)** Excitatory postsynaptic currents (EPSCs) evoked by optical stimulation **(D)** of ChR2 expressing terminals or by electrical stimulation **(F)** of parabrachial fibers were not affected by administration of nor-BNI (1 μM) (*p >* 0.05, paired *t*-test, compared to aCSF; optical, *n* = 8, electrical *n* = 6). Traces above graphs show individual examples. Optically evoked EPSCs were blocked by AP5 and CNQX. Yellow light (590 nm) stimulation as an inactive control did not evoke any responses. **(E,G)** Inhibitory postsynaptic currents (IPSCs) evoked by optical stimulation **(E)** and electrical stimulation **(G)** of parabrachial fibers were significantly increased by administration of nor-BNI (*p < 0.05, paired *t*-test, compared to predrug aCSF; optical, *n* = 8; electrical, *n* = 6). Traces show individual examples. Bicuculine as well as AP5 and CNQX blocked optical IPSCs. Yellow light stimulation served as a control. EPSCs and IPSCs were recorded at—70 and 0 mV, respectively. Bar histograms show means ± SEM. Symbols show values of individual neurons.

CRF and KOR have been shown to interact in the CeA in naïve animals (Ji and Neugebauer, 2020; Neugebauer et al., 2020; Hein et al., 2021). In brain slice physiology experiments we specifically targeted CRF neurons in the CeA (Section 5.7). Brain slices were obtained from morphine-primed rats within a 2–3 h window after the second RS (Timeline, Figure 6). We used a viral vector genetic approach to label CRF neurons in the CeA Section 5.7.2). CeA-CRF neurons were identified using mCherry fluorescent microscopy (Figure 6A). In response to depolarizing current injections, the majority (12 out of 14) of CRF neurons displayed a late firing phenotype (Figure 6B). Two neurons had a regular firing pattern. No difference in the effects of nor-BNI on these neuronal phenotypes was found, and therefore the data were pooled. Application of nor-BNI (1 μM by superfusion) decreased excitability of CeA-CRF neurons in the FPS model as compared to aCSF (*p* < 0.0001, F_1,144_ = 31.25, two-way ANOVA with Sidak’s posthoc test, *n* = 10, Figure 6C). No difference in resting membrane potential was found with nor-BNI application (*p* > 0.05, paired *t*-test, −58.9 + 1.9 mV, −59.2 + 1.7mV, *n* = 10, aCSF and nor-BNI respectively).

Optical (blue light) stimulation of glutamatergic terminals expressing ChR2 from the parabrachial nucleus (PB) and focal electrical stimulation of visually identified PB fibers dorsomedial to the CeA (Section 5.7.3) evoked synaptic responses. Stimulation parameters used in the current study were demonstrated previously to activate CeA neurons (Fu and Neugebauer, 2008; Sugimura et al., 2016). Optical as well as electrical activation of PB fibers evoked both excitatory and inhibitory postsynaptic currents (EPSCs and IPSCs) in all CeA-CRF neurons tested. Application of nor-BNI (1 μM, 15 min) did not change the amplitude of optically or electrically evoked EPSCs (*p* > 0.05, compared to vehicle aCSF, paired *t*-test, *n* = 8; Figures 6D,F) but significantly increased optically as well as electrically evoked IPSCs (*p <* 0.05, paired *t*-test, compared to predrug aCSF; *n* = 8; Figures 6E,G). EPSCs were blocked by glutamate receptor antagonists (AP5 and CNQX) and IPSCs were blocked by bicuculine and by AP5 and CNQX, indicating glutamate-driven GABAergic feedforward inhibition of CeA neurons from PB. Yellow light (590 nm) stimulation as an inactive control had no effect on CeA-CRF neurons.

**FIGURE 7:**
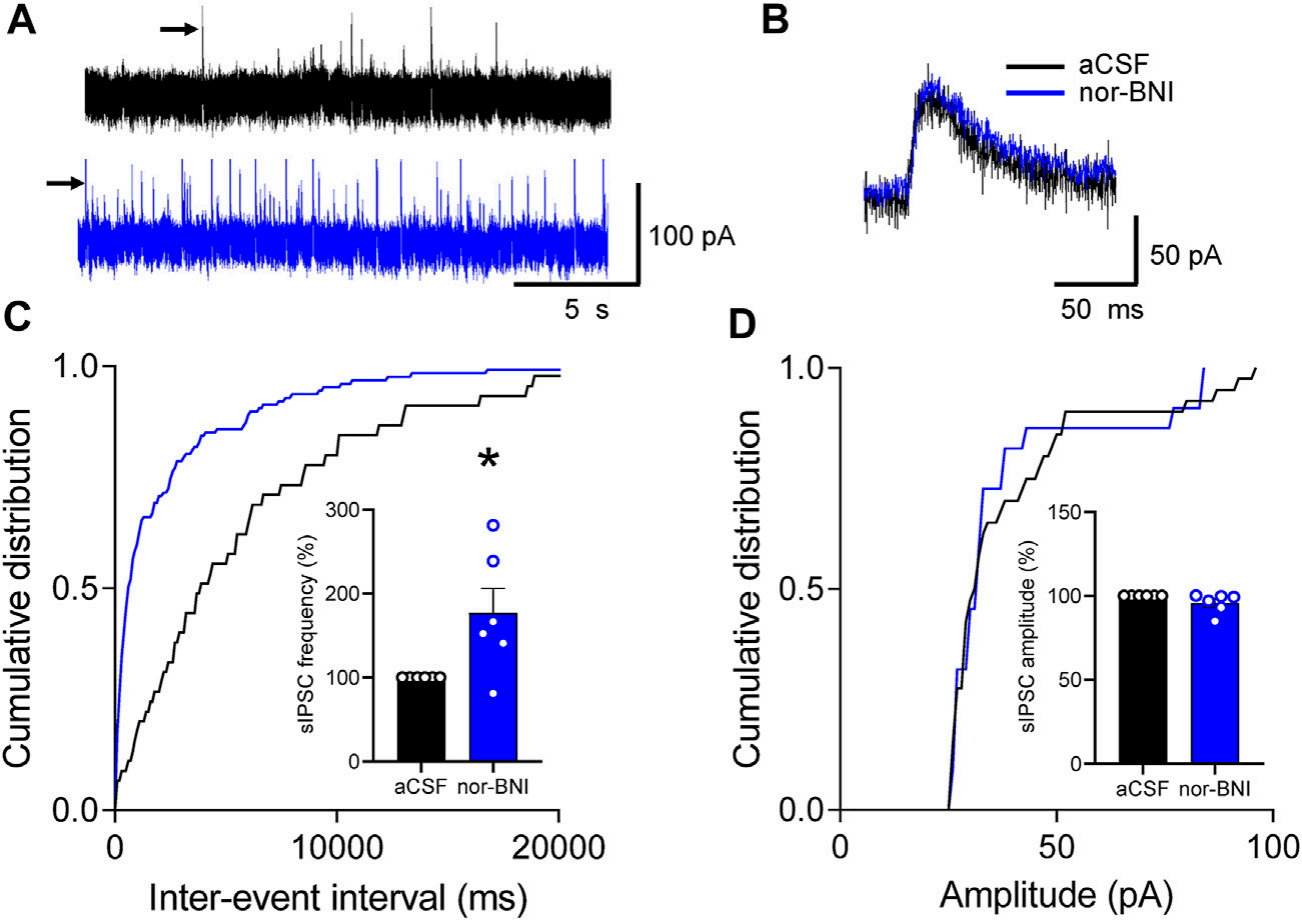
Effects of a KOR antagonist on spontaneous IPSCs (sIPSCs) in CRF-CeA neurons in a functional pain model. Recordings of visually identified CeA-CRF neurons were made in whole-cell configuration. **(A)** Voltage-clamped (at 0 mV) traces of sIPSCs during application of aCSF (vehicle) and nor-BNI (1 μM). **(B)** Expanded traces of individual sIPSCs (indicated by arrows in A) show that nor-BNI did not affect channel kinetics. **(C,D)** Analysis of frequency (inter-event interval) and amplitude of sIPSCs recorded in an individual neuron shows significantly increased sIPSC frequency by nor-BNI (*p < 0.05, paired *t*-test, compared to predrug aCSF, *n* = 6; **(C)**, but no significant effect on sIPSC amplitude (*p >* 0.05, paired *t*-test, compared to aCSF predrug control, *n* = 6; **(D)**. Bar histograms show means ± SEM. Symbols show values of individual neurons.

To determine the site of action of nor-BNI on inhibitory transmission, we analyzed spontaneous IPSCs (sIPSCs) in CeA-CRF neurons (Section 5.7.3). Superfusion of nor-BNI (1 μM, 15 min) significantly increased frequency, but not amplitude, of sIPSCs (*p <* 0.05, paired *t*-test, compared to aCSF, *n* = 6; Figures 7C,D).

The data show that blockade of KOR inhibition in CeA facilitates glutamate-driven synaptic feedforward inhibition from PB to CeA-CRF neurons in FPS, suggesting that endogenous activation of KOR results in decreased synaptic inhibition (disinhibition) of CeA-CRF neurons in this pain condition. KOR signaling in the CeA is pronociceptive in the FPS condition.

## 3 Discussion

The data presented here show for the first time that pharmacological blockade of KOR in the CeA with nor-BNI decreases pain-like and averse-affective behaviors by enhancing or restoring synaptic inhibition of CeA-CRF neurons in an FPS model, indicating a functional correlation between pain behaviors and KOR signaling in the amygdala in a functional pain syndrome (FPS) condition. The data further suggest that inhibitory control of CeA neurons containing CRF is impaired in the FPS condition while KOR blockade restores feedforward inhibition from the parabrachial nucleus as well as a spontaneous inhibitory tone in CeA-CRF neurons.

There is a need to develop preclinical models that are relevant to human FPS. We therefore adapted the novel hyperalgesic priming model in uninjured rats that may help understand mechanisms of the transformation of episodic pain to chronic pain. Morphine administered for 1 week produced a reversible hypersensitivity to innocuous tactile stimuli (transient central sensitization). In morphine-treated rats, exposure to restraint stress (RS) reinstated tactile allodynia. Stress is most often associated with aversive states that activate CRF neurons, which in turn can promote the release of dynorphin and engage the KOR system in the amygdala and other limbic brain areas (Koob, 1999; Land et al., 2008; Bruchas et al., 2009; Bruchas et al., 2010; Smith et al., 2012; Lalanne et al., 2014; Crowley et al., 2016; Tejeda et al., 2017).

This study focused on KOR actions in the amygdala because of evidence linking KOR in the amygdala to stress responses, aversive behaviors, and anxiety under normal conditions (Land et al., 2008; Bruchas et al., 2010; Smith et al., 2012; Crowley et al., 2016) and to aversive behaviors in stress-induced FPS models (Xie et al., 2017; Nation et al., 2018; Phelps et al., 2019). Particularly high expression levels of KOR are found in the CeA (Cahill et al., 2014). The amygdala is critically involved in emotional-affective behaviors and in disorders such as anxiety, depression and substance use disorders (Phelps and LeDoux, 2005; Apkarian et al., 2013; Koob and Schulkin, 2019). The amygdala plays an important role in affective and aversive aspects of pain, and the CeA output system is involved in pain control and modulation (Neugebauer et al., 2004; Thompson and Neugebauer, 2019; Neugebauer, 2020). Systemic or intra-CeA administration of a selective KOR agonist increased emotional responses (vocalizations), anxiety-like behaviors (EPM, open field test), and avoidance behaviors under normal conditions (Hein et al., 2021). Systemic application of a KOR agonist also produced place aversion behaviors in a neuropathic pain model (Liu et al., 2019).

Mechanisms of CeA KOR signaling in FPS-related conditions are only beginning to emerge. Recent evidence suggests that KOR signaling in the CeA promotes averse-affective behaviors in pain conditions triggered by stressors. Blockade of KOR with intra-amygdalar or with systemic application of nor-BNI prevented the development of pain-like behaviors in a stress-induced FPS model had no effect in control mice animals (Nation et al., 2018; Kopruszinski et al., 2021). Here we show that intra-CeA administration of nor-BNI decreased emotional responses (audible and ultrasonic vocalizations), anxiety-like behaviors (EPM), and hypersensitivity in the dynamic von Frey test but not with tonic compression of the tissue similar to the paw pressure test. This finding is consistent with previous study showing that KOR signaling in the CeA affects predominantly averse-affective rather than sensory aspects of pain (Navratilova et al., 2019; Hein et al., 2021). KOR antagonists were suggested previously as a new strategy for stress-related FPS management (Knoll and Carlezon, 2010; Nation et al., 2018). Our behavioral data support potential therapeutic benefits of KOR antagonists in FPS by mitigating averse-affective and pain-like behaviors. Nor-BNI is a highly selective KOR antagonist *in vitro* but systemic administration of nor-BNI has been reported to non-specifically antagonize mu and delta opioid receptors with short-term application while selectively blocking KOR with long-term application (Endoh et al., 1992; Horan et al., 1992; Bruchas et al., 2007). However, pretreatment with systemic nor-BNI 1 h prior to bright light stress blocked stress-induced allodynia, which could also be induced by a KOR agonist (U69593), suggesting blockade of KOR with short-term nor-BNI application (Xie et al., 2017). In our study, we tested nor-BNI *in vitro* and found beneficial inhibitory effects on CeA neurons, and in the behavioral experiments, nor-BNI was not given systemically but delivered directly into the amygdala by microdialysis for at least 15 min prior and during testing. Contribution of the blockade of other opioid receptors by nor-BNI to the observed behavioral outcomes seems therefore unlikely.

Our mechanistic study provides new insights into the action of KOR in FPS. The synaptic and cellular mechanisms of KOR signaling in CeA are not well understood, and we addressed this knowledge gap here. Electrophysiological studies in anesthetized animals have reported increased activity of individual CeA neurons evoked by innocuous and noxious stimulation in a model of arthritic pain (Neugebauer and Li, 2003; Li and Neugebauer, 2004; Ji and Neugebauer, 2007; Ji and Neugebauer, 2009) and in a neuropathic pain model (Goncalves and Dickenson, 2012; Ji et al., 2017; Ji and Neugebauer, 2019). So-called “multireceptive” CeA neurons are characterized by their stronger activation by noxious than innocuous stimulation; they may serve the integration and evaluation of sensory-affective processing in the context of pain (Neugebauer et al., 2004). Amygdala (CeA) neuronal plasticity in an FPS model remains to be determined. The systems physiology data in this study show that KOR blockade by intra-CeA microdialysis of nor-BNI inhibits spontaneous firing and stimulus-evoked responsiveness of these CeA neurons in an FPS model. We focused on the right CeA because previous electrophysiological and biochemical evidence suggests hemispheric lateralization of KOR signaling (Nation et al., 2018; Phelps et al., 2019; Navratilova et al., 2020) and pain-related function (Carrasquillo and Gereau, 2008; Ji and Neugebauer, 2009; Goncalves and Dickenson, 2012; Liu et al., 2019) to the right CeA.

This study on KOR also advances our knowledge about pain-related functions of amygdala CRF neurons. The CeA is comprised of diverse GABAergic neuronal populations expressing several peptides, including CRF, somatostatin, protein kinase C-delta (PKCδ), and neurotensin (McCullough et al., 2018; Neugebauer et al., 2020). These distinct neuronal populations differentially modulate diverse behaviors, including fear and pain-related behaviors (Haubensak et al., 2010; Wilson et al., 2019; Neugebauer et al., 2020). The amygdala CRF system has been implicated in stress, fear, anxiety, and depression (Gafford and Ressler, 2016; Pomrenze et al., 2019a; Pliota et al., 2020) and is also recognized as an important player in pain-related plasticity and behavior (Fu and Neugebauer, 2008; Neugebauer, 2015; Ji and Neugebauer, 2019; Mazzitelli et al., 2021). Upregulation of CRF expression levels in the CeA produced mechanical and visceral hypersensitivity, which was blocked by CRF knockdown (Johnson et al., 2015). Our previous electrophysiology study in brain slices found that a KOR antagonist increased synaptic inhibition and decreased synaptically evoked spiking of CeA neurons in a neuropathic pain model, and the compromised feedforward inhibition was could be restored by KOR blockade (Navratilova et al., 2019). That study did not identify the cell type of CeA neurons but we suggested that they might be CRF neurons based on their location and firing properties. In the current study, we specifically focused on identified CeA-CRF neurons because a majority of them co-express dynorphin (Marchant et al., 2007) and CeA-CRF neurons from widespread projections to brain regions involved in the modulation of averse-affective behaviors (Marcilhac and Siaud, 1997). KOR expression is particularly high in the CeA (Mansour et al., 1995; Le Merrer et al., 2009; Cahill et al., 2014), and stress-related KOR phosphorylation in the amygdala (Bruchas et al., 2009; Xie et al., 2017) has been associated with dynorphin actions downstream of CRF signaling. Again, we studied interactions of KOR signaling and CRF neurons in the right amygdala (CeA) because blockade of KOR in the right, but not left, has been shown to restore diffuse noxious inhibitory control (DNIC), a descending pain control mechanism, which is lost in stress-induced pain conditions (Nation et al., 2018).

Recording identified CeA-CRF neurons in brain slice electrophysiology experiments, we tested the hypothesis that blockade of KOR would restore feedforward inhibitory transmission and, as a consequence, reduce firing of CeA-CRF neurons. The CeA receives sensory nociceptive information from the spinal cord and brainstem through the spino-parabrachio-amygdala pathway from the PB (Jhamandas et al., 1996; Gauriau and Bernard, 2002; Neugebauer, 2015; Sugimura et al., 2016). Activation of PB neurons produces monosynaptic excitation and also engages glutamate-driven polysynaptic inhibition in the CeA by targeting GABAergic neurons in the intercalated cell mass and perhaps PKCδ neurons in the laterocapsular subdivision of the CeA (Neugebauer, 2020). Our slice physiology experiments demonstrate for the first time that a selective KOR antagonist restored synaptic inhibition of CeA-CRF neurons in a rat model of FPS. Synaptic responses were evoked by electrical activation of visually-identifiable presumed PB input or by selective optical (470 nm blue light) activation of ChR2 expressing glutamatergic PB terminals in the CeA. Monosynaptic excitatory (EPSCs) and polysynaptic glutamate-driven inhibitory (IPSC, feedforward inhibition) responses were evoked by activation of PB fibers. Yellow light served as a negative control and did not evoke any synaptic responses. Glutamate-driven feedforward inhibition of CeA-CRF neurons was confirmed by the blockade with a GABA_A_ receptor antagonist (bicuculline) and with NMDA and AMPA receptor antagonists (AP5 and CNQX, respectively). Previously we showed that a KOR agonist (U-69,593) induced disinhibition and increased neuronal activity of CeA-CRF-CeA neurons in naïve rats (Hein et al., 2021), and thus we hypothesized that endogenous KOR activation of CeA-CRF neurons would produce similar disinhibitory effects in the FPS model, resulting in pain behaviors. Interestingly, we found no effect of KOR blockade on excitatory transmission in CeA-CRF neurons similar to our previous study (Hein et al., 2021), arguing against a tonic KOR tone modulating CeA activity and pain behaviors. Our previous study (Hein et al., 2021) showed an exclusive effect of a KOR agonist on inhibitory, but not excitatory, transmission, which is consistent with KOR localization and action on GABAergic interneurons that project to CeA output neurons such as CRF neurons. Also, a KOR agonist did not modulate PB-evoked EPSCs in a subset of unidentified laterocapsular CeA neurons, though effects on inhibitory synaptic transmission were not explored in that study (Kissiwaa et al., 2020).

The presence of sIPSCs in CRF neurons in the FPS model suggests a tonic inhibitory tone, which was strongly enhanced by KOR antagonist in the current study. Most likely nor-BNI acts presynaptically, because in our previous study KOR activation decreased frequency of mIPSCs (in TTX). Impaired synaptic inhibition may be a side effect of endogenous opioid release that contributes to averse-affective behaviors observed in our FPS model as well as with exogenous activation in naïve rats (Hein et al., 2021). Restoring or enhancing impaired synaptic inhibition of CeA-CRF neurons would inhibit pain behaviors. Inhibitory interneurons that are activated by KOR blockade may include other neuronal cell types in CeA (PKCδ, somatostatin, and neurotensin) or intercalated cells and may contribute to increased sIPSCs in CeA-CRF neurons observed in this study. Nor-BNI also increased inhibitory transmission in the medial CeA (Gilpin et al., 2014), which might have contributed to the increased sIPSCs observed here, but these neurons do not receive monosynaptic parabrachial input, which is a major source of nociceptive information to the CeA.

## 4 Conclusion

The present study demonstrates that inhibitory control of CeA-CRF neurons is impaired in an FPS model. Here we show for the first time that KOR blockade in the CeA mitigates primarily averse-affective behaviors by restoring synaptic inhibition of CeA-CRF neurons. Blockade of KOR increased feedforward synaptic inhibition that was driven by glutamatergic PB input to the CeA. Data suggest that KOR antagonists could have beneficial effects in the management of FPS conditions.

## 5 Materials and Methods

### 5.1 Animals

Transgenic Crh-Cre and wild type male rats on Wistar background (8–11 weeks old, 200 g–350 g at time of testing) were housed in a temperature-controlled environment under a 12 h light/12 h dark cycle and had *ad libitum* access to food and water. Genetically modified Crh-Cre rats express Cre-dependent recombinase exclusively in CRF containing neurons (Pomrenze et al., 2015) allowing expression of fluorophores for visualization. Initial breeding pairs of Crh-Cre rats were kindly provided by Dr. Robert Messing (UT, Austin). On the day of the experiment, rats were allowed to acclimate habituate to the laboratory for at least 1 h. All experimental procedures were approved by the Institutional Animal Care and Use Committee (IACUC) at TTUHSC and were conducted in accordance with the guidelines of the National Institutes of Health and the International Association for the Study of Pain. All behavioral studies were performed with the experimenter blinded to the treatment conditions. Electrophysiological recordings were performed independently by three different researchers and the data were pooled for the final analysis.

### 5.2 Experimental Protocol

Hyperalgesic priming strategy with morphine followed by exposure to repeated restrained stress was used to induce the FPS model in all rats studied (Section 2.3). The effect of a selective KOR antagonist (nor-Binaltorphimine, nor-BNI, Tocris Bioscience, Bio-Techne Corporation, Minneapolis, MN) (Portoghese et al., 1987; Kishioka et al., 2013) was studied. In behavioral experiments, reverse microdialysis was used to deliver aCSF or nor-BNI into the CeA; Section 5.4). Behavioral assays were done 15 min after the start of microdialysis (Section 5.5). In systems electrophysiology experiments (Section 5.6), aCSF and nor-BNI were also administered into CeA by microdialysis. Simultaneous extracellular recordings tested effects of nor-BNI on mechanically evoked (innocuous and noxious) and background activity of CeA neurons (Section 5.6). In brain slice physiology experiments (Section 5.7), we took advantage of the genetic labeling technique to study the effects of nor-BNI in the population of CRF neurons in CeA (CeA-CRF) as was done in our lab previously (Hein et al., 2021). Spontaneous activity and optogenetically or electrically evoked synaptic activity from parabrachial nucleus (PB) were measured in CeA-CRF neurons. In all experiments, nor-BNI applications and neuronal recordings were done in the right CeA because of strong evidence for lateralization of KOR signaling and pain processing to the right CeA (Ji and Neugebauer, 2009; Nation et al., 2018; Phelps et al., 2019).

### 5.3 Functional Pain Syndrome Model

A novel, injury-free FPS model was initially developed at the University of Arizona (Tucson, AZ, United States) (Nation et al., 2018; Kopruszinski et al., 2021). A two-stage approach was implemented for the induction of FPS: 1) hyperalgesic priming with morphine and 2) repeated restraint stress (RS). On day 1, anesthesia was induced with 5% and maintained with 2% isoflurane (Nation precision vaporizer, Harvard Apparatus, Holliston, MA, United States) for the subcutaneous implantation of osmotic minipumps (Model 2001; Alzet, Cupertino, CA, United States) into the interscapular region to deliver vehicle (0.9% saline) or morphine sulfate (Sigma-Aldrich, St. Louis, MO, United States; 7.68 mg/kg/day) at a rate of 1 μl/h for 7 days. On day 7, rats were briefly anesthetized with isoflurane to remove osmotic pumps. On day 20 and day 21, rats received 1 h long RS. Rats were placed in rodent cone restrainers (DecapiCone, Braintree Scientific Inc. Braintree, MA, United States). Cones were squeezed closed behind the body forcing the nose of the animal towards the narrow open end. Rubber bands wrapped behind the animal’s body allowed the tail to protrude from the cone. Animals were observed continuously during the stress exposure. Final behavioral tests and electrophysiological recordings were conducted within a 2–3 h window after second RS.

### 5.4 Drug Application Into Central Nucleus of Amygdala of Awake Animals

Drug administration into the CeA by reverse microdialysis is a well-established technique in our laboratory (Thompson et al., 2015; Cragg et al., 2016; Kiritoshi et al., 2016; Mazzitelli and Neugebauer, 2019; Hein et al., 2021). A guide cannula for microdialysis (CMA/Microdialysis, Solna, Sweden) was implanted 4–5 days prior to the experiment to give animals enough time to heal and accommodate the cannula. Rats were anesthetized with isoflurane and their head was fixed in a stereotaxic frame (David Kopf Instruments, Tujunga, CA). A small drill hole was made in the scull and a guide cannula was implanted into the right CeA using the following coordinates: 2.5 mm caudal to bregma, 4.3 mm lateral to midline, and 6.5 mm deep (Paxinos and Watson, 1998). The cannula was securely attached to the skull with Metabond (Parkell, Edgewood, NY, United States) or dental cement (Plastic One, Roanoke, VA). Topical antibiotic (Bacitracin) was applied daily to prevent infection. For drug administration into the CeA, a microdialysis probe (CMA/Microdialysis) was inserted into the guide cannula on the day of the experiment. The probe was connected to a programmable syringe infusion pump (Harvard Apparatus) with polyethylene tubing and protruded by 1 mm from the guide cannula, reaching a target depth of 7.5 mm. Nor-BNI (Tocris Bioscience, Bio-Techne Corporation, Minneapolis, MN) or aCSF (Section 5.7.1 for composition of aCSF) was administered at a rate of 5 μl/min. The nor-BNI stock solution was diluted in aCSF to the intended concentration (100 μM), which 100 times higher than the target concentration achieved in the tissue (1 μM) due to the concentration gradient across the membrane of the microdialysis probe and drug diffusion in the brain tissue as described before (Kiritoshi et al., 2016; Thompson et al., 2018; Mazzitelli and Neugebauer, 2019). Administration of aCSF always preceded the infusion of nor-BNI. To establish equilibrium in the brain tissue, aCSF and nor-BNI were continuously administered for at least 15 min.

### 5.5 Behaviors

#### 5.5.1 Mechanosensitivity

Mechanosensitivity to tactile stimuli was measured using an electronic von Frey anesthesiometer (IITC Life Science, Woodland Hills, CA). The rigid tip of anesthesiometer was applied with increasing force to the base of the third or fourth toe of the left hind paw until a paw withdrawal response (nocifensive reflex) was provoked. The force required to evoke a withdrawal reflex was recorded automatically (in grams) as the mechanical threshold. Three consecutive measurements were averaged. As the least invasive, this test was always performed prior to any other behavioral assay. After the vocalization assay (Section 5.5.2), mechanical thresholds were also measured using a calibrated forceps (tip area of 30 mm^2^) with a force transducer to compress the left hind paw with continuously increasing force until a reflex response was evoked, which was displayed on an LCD screen and recorded automatically as the mechanical threshold (g/30 mm^2^). Three measurements during aCSF or nor-BNI application were averaged to calculate nocifensive reflex thresholds.

#### 5.5.2 Affective Behaviors

Vocalizations in the audible (20 Hz-16 kHz) and ultrasonic (25 ± 4 kHz) ranges were recorded using a microphone and a bat detector, respectively, as in our previous studies (Han et al., 2005; Kiritoshi et al., 2016; Mazzitelli and Neugebauer, 2019; Presto et al., 2021; Mazzitelli et al., 2022). Under brief anesthesia with isoflurane, rats were placed in a custom-designed plexiglass chamber that permitted access to the paws for mechanical stimulation (U.S. Patent 7,213,538). Animals recovered quickly and were habituated to the chamber for at least 10 min. A calibrated forceps was used to apply noxious stimuli (1000–1500 g/30 mm^2^, 15 s) to the left hindpaw to evoke vocalizations. Signals from the microphone and bat detector were digitized and recorded for 1 min after the start of mechanical stimulation with Ultravox interface (UltraVox2, Noldus Information Technology, Leesburg, VA). Vocalizations were measured twice in the same animal: during aCSF microdialysis and after 15–30 min of nor-BNI microdialysis.

#### 5.5.3 Anxiety-Like Behaviors

Anxiety-like behaviors were assessed in the elevated plus maze (EPM, Noldus Information Technologies) as described previously by our group (Cragg et al., 2016; Ji et al., 2018; Mazzitelli et al., 2022). Movement in the EPM was videotracked for 5 min with EthoVision software (Noldus Information Technology). EPM contains elevated (60 cm above the floor) two open arms and two enclosed arms (10 × 50 cm each) that are connected by a central area (10 × 10 cm). Each trial started by placing the animal in the center area facing an open arm. Vehicle (aCSF) and nor-BNI were delivered by microdialysis 15 min before and during the EPM (5 min) test. Open arm choice (percentage of open arm duration/[open + close arms duration]), open arm entries (percentage of the number of entrees into the open arms over the total number of entries) and locomotor activity (total distance traveled in cm) were measured for the duration of the assay (5 min). Avoidance of the open arms of the EPM was interpreted as anxiety-like behavior.

### 5.6 Systems Electrophysiology

#### 5.6.1 Extracellular Unit Recording

Using glass insulated carbon fiber microelectrodes (4–6 MΩ), single-unit recordings were made from multireceptive CeA neurons in the as described previously (Ji et al., 2018; Ji and Neugebauer, 2020). Briefly, anesthesia was induced and maintained with isoflurane (3–4% and 2%, respectively; Harvard Apparatus). Body core temperature was maintained at 37°C. The animal was placed in a stereotaxic frame (David Kopf Instruments), and a craniotomy was performed at the level of the *sutura frontoparietalis* for the insertion of the recording electrode and microdialysis probe for drug or vehicle administration (Section 5.4). The following coordinates were used for stereotaxic recordings of CeA neurons: 2.3–2.8 mm caudal to bregma, 3.8–4.2 mm lateral to midline, 7–8 mm deep (Paxinos and Watson, 1998). The recorded activity was amplified, filtered (bandwidth 300 Hz—3 kHz) and digitized using 1401 Plus interface (Cambridge Electronic Design, Cambridge, UK). Action potentials were sorted, analyzed and stored using Spike two software (Cambridge Electronic Design). Peristimulus rate histograms were created on-line.

CeA neurons were identified by their background activity and responses to innocuous (100 g/6 mm^2^) and noxious (500 g/6 mm^2^) mechanical stimulation of the contralateral hindpaw (compression with a calibrated forceps; Section 5.5.2). Only neurons that were activated more strongly by noxious than innocuous stimuli were selected for the analyses. Background neuronal activity in the absence of intentional stimulation was recorded for 10 min and measured as spikes/s. Brief (15 s) innocuous and noxious stimuli (15 s inter-stimulus interval) were applied to the hindpaw. Net evoked activity was measured by subtracting activity during stimulation from background activity preceding the stimulus. At the end of each experiment a 250 μA DC was passed through the recording microelectrode for 3 min to mark the location of recording site (Section 5.8).

#### 5.6.2 Intra-Amygdala Drug Application by Microdialysis

A microdialysis probe (CMA/Microdialysis) for aCSF or nor-BNI administration was inserted into the right CeA 1 h before single-unit recordings. To allow the simultaneous positioning of the microdialysis probe (Section 5.4) and single unit recordings in the right CeA, the probe was inserted at a 5^o^ angle using the following coordinates: 1.8–2.3 mm caudal to bregma, 4.0–4.5 mm lateral to midline, 8.0 mm deep) (Paxinos and Watson, 1998). Vehicle (aCSF; Section 5.7.1 for composition) was continuously perfused through the microdialysis probe with an infusion pump (Harvard Apparatus, 5 μl/min) during the search and recording of neurons. Nor-BNI (100 μM in the microdialysis probe as in the behavioral experiments; Section 5.5) was administered for at least 15 min before testing to establish equilibrium in the tissue.

### 5.7 Brain Slice Electrophysiology

#### 5.7.1 Brain Slice Preparation

Rats were decapitated, brains rapidly removed and immersed in oxygenated ice-cold sucrose-based slicing solution containing the following (in mM): 87 NaCl, 75 sucrose, 25 glucose, 5 KCl, 21 MgCl2, 0.5 CaCl2, and 1.25 NaH2PO4. Coronal slices (400 μm) were prepared using a Vibratome (VT1200S, Leica Biosystems, Nussloch, Germany). Slices containing the right CeA were incubated in oxygenated aCSF at room temperature (21°C) for at least 1 h before patch recordings. Recording aCSF contained the following (in mM): 117 NaCl, 4.7 KCl, 1.2 NaH_2_PO_4_, 2.5 CaCl_2_, 1.2, MgCl_2_, 25 NaHCO_3_, and 11 glucose. For whole-cell patch recording, a brain slice containing CeA was transferred to the recording chamber and superfused with oxygenated aCSF (31°C) at 2 ml/min. Two brain slices per animal were used and only one neuron was recorded in each slice because of long-lasting pharmacokinetics of nor-BNI (Kishioka et al., 2013).

#### 5.7.2 Identification of Central Nucleus of Amygdala-Corticotropin Releasing Factor Neurons in Brain Slices

Viral vector AAV5-EF1α-DIO-mCherry was injected into the right CeA (1.8–2.3 mm caudal to bregma, 4.0–4.5 mm lateral to midline, 7.5–8.0 mm deep (Paxinos and Watson, 1998) of Crh-Cre rats (Section 5.1) 4–5 weeks before brain slices were obtained to allow for viral vector expression and labeling of CRF neurons as described before (Hein et al., 2021) (Figure 1A). LED light source (X-Cite 120 Led Boost) with an ET-DS Red filter (ET545/30x, Chroma Technology Corp., Bellows Falls, VT) of an Olympus microscope (BX51, Olympus, Waltham, MA) was used to visualize mCherry-expressing CRF neurons in the CeA.

#### 5.7.3 Patch-Clamp Recordings of Corticotropin Releasing Factor-Central Nucleus of Amygdala Neurons

Whole-cell patch-clamp recordings were obtained from visually identified mCherry-positive CRF neuros in the CeA (Figure 1A) using fluorescence microscopy (Section 5.7.2) as described previously (Thompson et al., 2018; Navratilova et al., 2019; Hein et al., 2021). Borosilicate glass recording electrodes (4–8 MΩ tip resistance) were filled with an intracellular solution containing (in mM): 122 K-gluconate, 5 NaCl, 0.3 CaCl2, 2 MgCl2, 1 EGTA, 10 HEPES, 5 Na2-ATP, and 0.4 Na3-GTP; pH was adjusted to 7.2–7.3 and osmolarity to 280 mOsm/kg. Signals were amplified with Multiclamp 700B amplifier (Axon Instruments), digitized using a Digidata 1550B interface (Axon Instruments, Molecular Devices, San Jose, CA), and pClamp11 software (Axon Instruments). Access resistance was continuously monitored during recording and if changed >20%, the neuron was discarded.

*Excitability* was measured in current-clamp. Action potentials were evoked by depolarizing current steps (0.5 s) of increasing amplitude (25 pA) from the resting membrane potential. Neuronal excitability was measured from frequency-current (F–I) functions.

*Synaptic transmission* was measured in voltage-clamp using optical and electrical stimulation of fibers originating from external parabrachial (PB) nucleus. Excitatory and inhibitory synaptic currents (EPSCs and IPSCs) were recorded in voltage-clamp at −70 and 0 mV, respectively.

For optogenetic stimulation, an AAV vector encoding channel rhodopsin 2 (ChR2) under the promoter of CaMKII (rAAV5/CaMKIIa-ChR2(H134R)-eYFP, University of North Carolina, Chapel Hill) was administered stereotaxically into the right PB using the following coordinates: 15^o^ anteroposterior angle, 6.6–6.5 mm caudal to bregma, 2.2–2.3 lateral, 7.4–7.1 mm deep (Paxinos and Watson, 1998; Sugimura et al., 2016) (Figure 1B). Slices were collected after 4 weeks to ensure viral expression. ChR2-expressing fibers were visualized in the lateral and more strongly in the capsular division of the CeA (Figure 1A). Blue light pulses (470 nm, 5 ms) were applied to activate ChR2-expressing fibers from the PB by passing broad spectrum LED light (X-Cite 120 led Boost, Excelitas Technologies Corp.) through a blue filter (ET470/40 × 470 nm, Chroma Technology Corp.) and microscope objective. Yellow light (595 nm, 5 ms) filtered by ET585/20 m (Chroma Technology Corp.) served as an inactive control.

For focal electrical synaptic stimulation (0.15 ms square-wave pulse delivered by A365 isolation unit; World Precision Instruments) a concentric bipolar electrode (David Kopf Instruments) was positioned on the visually identified fiber tract dorsomedial to CeA and lateral to caudate putamen as previously described (Fu and Neugebauer, 2008; Ren et al., 2013; Navratilova et al., 2019).

*Spontaneous IPSCs* (sIPSCs) were recorded in voltage clamp by holding cells at 0 mV as described previously (Ji et al., 2010; Kiritoshi et al., 2016). Frequency and amplitude distributions in 5 min traces were analyzed for using MiniAnalysis program 6.0.7 (Synaptosoft, Decatur, GA). Background noise (root mean square, RMS) was computed for each trace and event detection threshold was set to 4 times RMS. All detected events were visually inspected. Frequency and amplitude of sIPSCs were measured 5–10 min before (in aCSF) and 10–15 min during nor-BNI application.

EPSCs and IPSCs were confirmed by their sensitivity to DL-2-Amino-5-phosphonopentanoic acid (DL-AP5, 50 µM) and 6-cyano-7-nitroquinoxaline-2,3-dione disodium salt hydrate (CNQX, 10 µM) or bicuculline (30 µM), respectively.

The nor-BNI stock solution was diluted in aCSF to 1 μM (Hein et al., 2021). All drugs were obtained from Tocris Bioscience.

### 5.8 Histological Verification of Drug Injection and Recording Sites

ChR2-YFP injection sites into parabrachial (PB) nucleus and expression in PB-projecting terminals in CeA was confirmed with confocal (FV3000, Olympus, Center Valley, PA) or fluorescent microscopy during patch recordings. (Figures 2A,B). Locations of the tips of the microdialysis probes and recording electrodes from *in vivo* electrophysiology were verified histologically after experiments (Figure 2C) with reference to a brain atlas (Paxinos and Watson, 1998). Brains were fixed overnight in 4% paraformaldehyde, cryoprotected in 30% sucrose, sectioned (30 μm) on a cryostat and analyzed with bright field microscope.

### 5.9 Data and Statistical Analysis

All values are presented as means ± SEM. GraphPad Prism 7.0 software (Graph-Pad Software, San Diego, CA) was used for statistical analyses. Statistical significance was accepted at the level *p* < 0.05. Two-way or one-way analysis of variance (ANOVA, repeated measures if appropriate) with appropriate post-hoc tests was used for multiple comparisons. Two datasets (before-during drug application) were compared using *t*-statistics (paired or unpaired *t*-tests, as appropriate). Parametric tests were used on data that passed Shapiro-Wilk and Kolmogorov-Smirnov normality tests (Graph-Pad software). Group sizes were estimated a priori by conducting power analysis on existing data to predict the effect size with a statistical significance at an alpha of 0.05 for a power of 80%.

## Data Availability

The original contributions presented in the study are included in the article/supplementary material, further inquiries can be directed to the corresponding author.
